# Efficacy and safety of anlotinib in patients with unresectable or metastatic bone sarcoma: A retrospective multiple institution study

**DOI:** 10.1002/cam4.4286

**Published:** 2021-09-26

**Authors:** Zhiyong Liu, Songtao Gao, Liangyu Zhu, Jiaqiang Wang, Peng Zhang, Po Li, Fan Zhang, Weitao Yao

**Affiliations:** ^1^ Department of Orthopedics The Affiliated Cancer Hospital of Zhengzhou University and Henan Cancer Hospital Zhengzhou Henan China; ^2^ Department of Orthopedics Henan Provincial People's Hospital and People's Hospital of Zhengzhou University Zhengzhou Henan China; ^3^ Department of Orthopedics Zhengzhou Orthopedics Hospital Zhengzhou Henan China

**Keywords:** anlotinib, bone sarcoma, progression‐free survival, safety

## Abstract

**Background:**

Tyrosine kinase inhibitors (TKIs) such as cabozantinib, regorafenib have demonstrated encouraging activity in prolonging progression‐free survival (PFS) in several bone sarcoma entities in prospective clinical trials. This retrospective study aims to analyze the safety and efficacy of anlotinib, a novel multi‐target TKI, in patients with locally unresectable or metastatic bone sarcoma at three institutions.

**Methods:**

Patients with advanced bone sarcoma administered anlotinib 12 mg once daily, 2 weeks on/1 week off, from June 2018 to June 2020, until disease progression or intolerance of treatment. The primary endpoints were objective response rate (ORR) and PFS.

**Results:**

Forty‐eight patients were analyzed: 27 have osteosarcoma, 9 have chondrosarcoma, 8 have Ewing's sarcoma, and 3 have chordoma. The median age was 24 years (range, 16–68 years), and the median number of prior regimens was 1 (range, 0–4). Until the final follow‐up, five patients obtained a partial response and while 24 achieved stable disease. The ORR in all patients was 10.4%, and the median PFS was 4.6 months, with a progression‐free rate (PFR) at 3 months and 6 months of 72.9% and 35.4%, respectively. The ORR and median PFS varied much among tumor subtypes. The most frequent grade 3–4 adverse events (AEs) were pneumothorax, hand‐foot syndrome, cholesterol elevation, hypertriglyceridemia, and fatigue. No patients died from anlotinib‐related AEs during the study period.

**Conclusions:**

Anlotinib may show promising antitumor activity in unresectable or metastatic bone sarcoma. The ORR and median PFS of anlotnib are similar to those of other targeted drugs in different subtypes of sarcomas. The AEs were generally mild and tolerated well. Further studies of anlotinib in selected subtypes of bone sarcoma are needed.

## Graphical abstract

Anlotinib may show promising antitumor activity in unresectable or metastatic bone sarcoma. The ORR and median PFS of anlotnib were similar to those of other targeted drugs in different subtypes of sarcomas. The AEs were generally mild and well tolerated. Further studies of anlotinib in selected subtypes of bone sarcoma are needed.

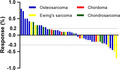

## 1 INTRODUCTION

Bone sarcoma is a collective term for heterogeneous primary malignant bone tumors, mainly osteosarcoma, Ewing's sarcoma, chondrosarcoma, and chordoma. The overall incidence is estimated at 0.8 per 100,000 person‐years.^1^, ^2^, ^3^ Osteosarcoma and Ewing's sarcoma occur frequently in children and adolescents and rank as the third leading cause of cancer‐related mortality in patients under the age of 20 years. The multimodal therapy, including radical surgery (and/or radiation) and multiagent chemotherapy, has dramatically improved the prognosis of patients, especially the extremity localized diseases, for which the 5‐year survival rate increases from 10%–20% to 60%–80%.^3^, ^4^ Chondrosarcoma and chordoma often affect adults 40–70 years old. In general, complete surgical resection is the standard therapy for patients with localized disease, and a high histological grade, presence of local recurrence, and metastatic disease are considered to be associated with unfavorable survival.^3^, ^5^ Patients with unresectable or metastatic bone sarcoma fare poorly because current treatment strategies, including chemotherapy and radiotherapy, could not control multifocal diseases durably. Those therapies often carry short‐term and long‐term unbearable toxicities.^6^ Hence, it is urgent to explore novel therapeutic approaches to manage this deadly disease more effectively.

Aberrant angiogenesis results in new blood vessel development playing crucial roles in the growth, invasion, and metastasis of numerous tumors, including bone sarcoma. Preclinical studies have revealed that vascular endothelial growth factor (VEGF) and vascular endothelial growth factor A (VEGF‐A) are detected in tumor lesions, and VEGF and VEGF‐A expression are positively correlated with pathological grade of bone sarcoma and negatively correlated with progression‐free survival (PFS). The discovery suggests that drugs which therapeutically block tyrosine kinase receptors or their ligands may play an essential role in advanced diseases.^7^ Multitarget tyrosine kinases inhibitors (TKIs), such as sorafenib, regorafenib, and cabozantinib, have demonstrated objective response rate (ORR) benefits and PFS superiority in advanced bone sarcoma.^8^, ^9^, ^10^ As a novel multi‐target TKI developed independently in China, anlotinib has shown in vitro and in vivo antitumor effect on several types of tumor models by blocking vascular endothelial growth factor receptor (VEGFR), fibroblast growth factor receptor (FGFR), platelet‐derived growth factor receptors (PDGFR), c‐Kit, and Ret simultaneously, and has a broad‐spectrum involvement in tumorigenesis and tumor angiogenesis.^11^ Clinical studies have revealed ORR and survival benefits and manageable adverse event profiles of anlotinib compared to placebo in several cancers, including non‐small cell lung cancer, and soft tissue sarcomas.^11^, ^12^ Additionally, anlotinib has shown promising activity in inhibiting osteosarcoma cell lines proliferation, and tumor growth, and metastasis in vitro and in vivo models through a variety of pathways.^13^ This suggests that anlotinib may be a reasonable option because there is no effective TKI approved against these advanced sarcomas in China.

Therefore, we hypothesized that anlotinib could be safely and efficiently administered to patients with advanced bone sarcoma, and patients with advanced diseases who had disease progression after standard therapy and began to receive anlotinib treatment in three institutions since June 2018. Here, we report the efficacy and safety of anlotinib in patients with unresectable or metastatic bone sarcoma objectively, and aim to determine whether anlotinib needs further study in advanced diseases in clinical practice.

## 2 MATERIALS AND METHODS

### 2.1 Patient population and data collection

The clinical data of patients diagnosed with progressive bone sarcoma not eligible for surgery at Henan Cancer Hospital, Henan Provincial People's Hospital, and Zhengzhou Orthopedics Hospital from June 2018 to December 2020 were retrospectively analyzed. The following eligibility criteria were applied: (1) 16 years of age or older; (2) Histologically confirmed bone sarcoma including osteosarcoma, Ewing's sarcoma, conventional chondrosarcoma, chordoma; (3) Have unresectable or metastatic tumor lesions which are not suitable for curative treatment; (4) Treated with at least one cycle of anlotinib treatment; (5) Performance status ranging from 0 to 2 according to the Eastern Cooperative Oncology Group (ECOG); (6) Adequate hematologic, hepatic, cardiac, and renal function; (7) At least one measurable tumor as defined by Response Evaluation Criteria in Solid Tumors (RECIST 1.1); (8) Normal or controlled blood pressure; and (9) Confirmed progressive disease based on standard imaging criteria within 6 months. Exclusion criteria include: (1) Have previously received other TKIs; and (2) Treated with anlotinib combined with other treatments, such as radiotherapy, chemotherapy, and immunotherapy. Clinical characteristics were recorded regularly throughout the study, including gender, age, histological characteristics, medication history, primary locations of sarcomas, metastatic sites, efficacy (ORR and PFS), and adverse events.

### 2.2 Therapeutic schemes

Patients were initially administrated anlotinib in 21‐day cycles (provided by Chia Tai Tin Ching Company), at a dose of 12 mg/day from days 1 to 14. Treatment was continued until diseases progressed or unacceptable AEs occurred. AEs were classified and graded using the National Cancer Institute Common Terminology Criteria for Adverse Events (NCI‐CTCAE), version 4.0. The dose was permitted to reduce or temporarily suspend according to patient tolerance. The initial dose of 12 mg/day anlotinib was allowed to be reduced to 10 mg and then to 8 mg, when AEs became tolerable, a higher dose could be given to patients again.

### 2.3 Assessment of safety and toxicity

Radiological assessment of target lesions by computed tomography (CT) scans or magnetic resonance imaging (MRI) was performed at baseline, every 6–8 weeks thereafter and if clinically indicated, and was analyzed according to RECIST (version 1.1). A dual primary endpoint of objective response rate during the study period and median PFS were assessed. The best responses in patients were recorded from the treatment initiation to the time of progression, and were further categorized into either complete response (CR), partial response (PR), stable disease (SD), or progressive disease (PD). ORR was defined as the percentage of patients who experienced CR and PR. PFS was calculated from the onset of treatment to the first documented disease progression or death, whichever occurred first. AEs were graded and recorded using NCI‐CTCAE, v 4.0.

### 2.4 Statistical analysis

Quantitative variables were reported in terms of the median (range) or frequency (percentage). PFS and the corresponding 95% confidence intervals (CIs) were performed by the Kaplan–Meier method. Statistical analyses were calculated using SPSS version 21.0 (IBM, Chicago, IL). Survival curves were generated using Prism 8.0 (GraphPad Software, La Jolla, CA).

## 3 RESULTS

### 3.1 Patient characteristics

Fifty‐one patients with unresectable or metastatic bone sarcoma treated with anlotinib were registered from June 2018 to December 2020. Three patients were lost to follow‐up, and 48 patients were finally included: 27 osteosarcoma patients, nine chondrosarcoma patients, eight Ewing's sarcoma patients, four chordoma patients. The baseline characteristics are summarized in Table 1. The median age was 24 years (range, 16–68 years), with 29 males and 19 females. The median number of prior therapeutic regimens was 1 (range, 0–4), 25 of 27 osteosarcoma patients, and five of nine chondrosarcoma patients, eight of eight Ewing's sarcoma patients, and one of four chordoma patients had received at least one line (range 1–4) of systemic chemotherapy before anlotinib treatment. Metastasis occurred in 37 patients (77.1%), including single lung metastasis in 29 (60.4%) patients, followed by multiple organ metastasis in nine (18.8%) patients. ECOG performance status was 0 or 1 for most patients. 28 (58.3%) bone sarcoma originated primarily from the extremities, nine (18.8%) from vertebra, and eight (16.7%) from pelvic girdle. Forty‐three (89.6%) patients had a history of primary resection.

**TABLE 1:** Baseline characteristics of patients

Baseline characteristics	Number of patients (*n*)	Percentage (%)
Age (years)		
Median	24	
Range	16–68	
Sex		
Male	29	60.4
Female	19	39.6
ECOG performance status		
0	26	54.2
1	13	27.1
2	9	18.8
Histology		
Osteosarcoma	27	56.3
Chondrosarcoma	9	18.8
Ewing's sarcoma	8	16.7
Chordoma	4	8.3
Primary tumor site		
Extremities	28	58.3
Vertebra	9	18.8
Pelvic girdle	8	16.7
Others	3	5.3
Distant metastases		
Lung only	29	60.4
Multiple organs	9	18.8
Radiotherapy history		
Yes	8	16.7
No	40	83.3
Surgery history		
Yes	43	89.6
No	5	10.4
Chemotherapy history		
Yes	39	81.3
No	9	18.8
Number of prior chemotherapy lines		
0	9	18.8
1	18	37.5
2	15	31.3
≥3	6	12.5

Abbreviations: ECOG, Eastern Cooperative Oncology Group; Others, Skull, Shoulder girdle.

Due to the discrepancy between the subtypes of bone sarcoma, patients had received different regimens before taking anlotinib treatment. Most osteosarcoma patients received standard chemotherapy including cisplatin, doxorubicin and/or ifosfamide and high‐dose methotrexate. Some patients were re‐challenged with docetaxel combined with gemcitabine, or a liposomal doxorubicin‐platinum‐based or ifosfamide‐based regimen. The Ewing's sarcoma patients had been treated with a three‐drug regimen of VDC (vincristine, doxorubicin, cyclophosphamide) or a five‐drug regimen of VDC‐IE (vincristine, doxorubicin, cyclophosphamide, etoposide and ifosfamide), the majority of whom received different treatment options of a cyclophosphamide and topotecan regimen, docetaxel and gemcitabine regimen, or high‐dose ifosfamide after tumor progression. Five of 13 patients with chondrosarcoma and chordoma had been given at least an anthracycline‐based chemotherapy.

### 3.2 Treatment

The median number of administered anlotinib cycles was 8 (range 1–29). At the time of the analysis (July 1, 2021), five patients continued undergoing anlotinib treatment, while the other 43 patients (89.6%) had stopped. The reasons for treatment discontinuation were disease progression and unacceptable AEs. Eight (16.7%) of 48 patients underwent dose reduction or treatment discontinuation due to AEs. A permanent dose reduction was required in four (8.3%) of 48 patients: to 10 mg/day in 2 (4.2%) patients, 8 mg/day in 2 (4.2%) patients, most of whom had been given three (range, 1–14) treatment cycles before treatment was discontinue because AEs occurred.

### 3.3 Efficacy

**FIGURE 1:**
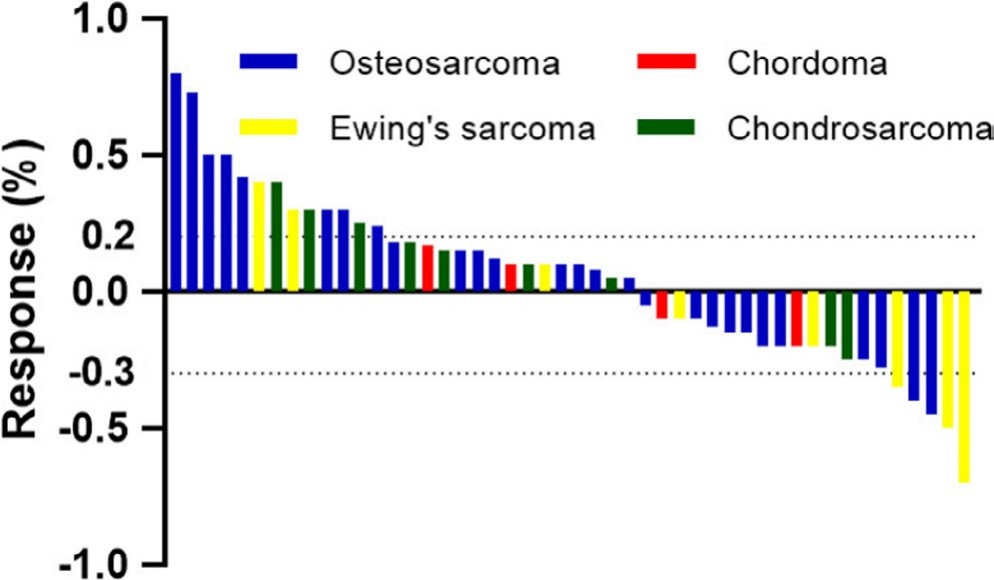
Waterfall plots for maximum changes in sizes of target lesions versus baseline during anlotinib treatment. The dashed lines represent the criteria for progressive disease (20% increase in target lesions size) and partial response (30% decrease in target lesions size)

Median follow‐up was 8 months (range, 3–20), although none had CR, five patients had PR, 30 patients obtained SD. As summarized in Table 2, the ORR was 10.4% (7.4% for osteosarcoma, 37.5% for Ewing's sarcoma, 0 for chondrosarcoma, and 0 for chordoma respectively). The response conditions are shown in Figure 1.

**TABLE 2:** Responses of various histological subtypes to treatment

	CR	PR	SD	PD	ORR	mPFS	3 m‐PFR	6 m‐PFR
Osteosarcoma	0	2	16	9	7.4%	4.7 m	75%	37%
Ewing's sarcoma	0	3	3	2	37.5%	6.7 m	75%	50%
Chondrosarcoma	0	0	7	2	0	4.2 m	66.7%	33.3%
Chordoma	0	0	3	1	0	3.2 m	50%	25%
Total	0	5	29	14	10.4%	4.6 m	70.8%	35.4%

Abbreviations: 3 m‐PFR, progression‐free rate (PFR) at 3 months; 6 m‐PFR, progression‐free rate (PFR) at 6 months; CR, complete response; mPFS, median progressive‐free survival; ORR, objective response rate; PD, progressive disease; PR, partial response; SD, stable disease.

**FIGURE 2:**
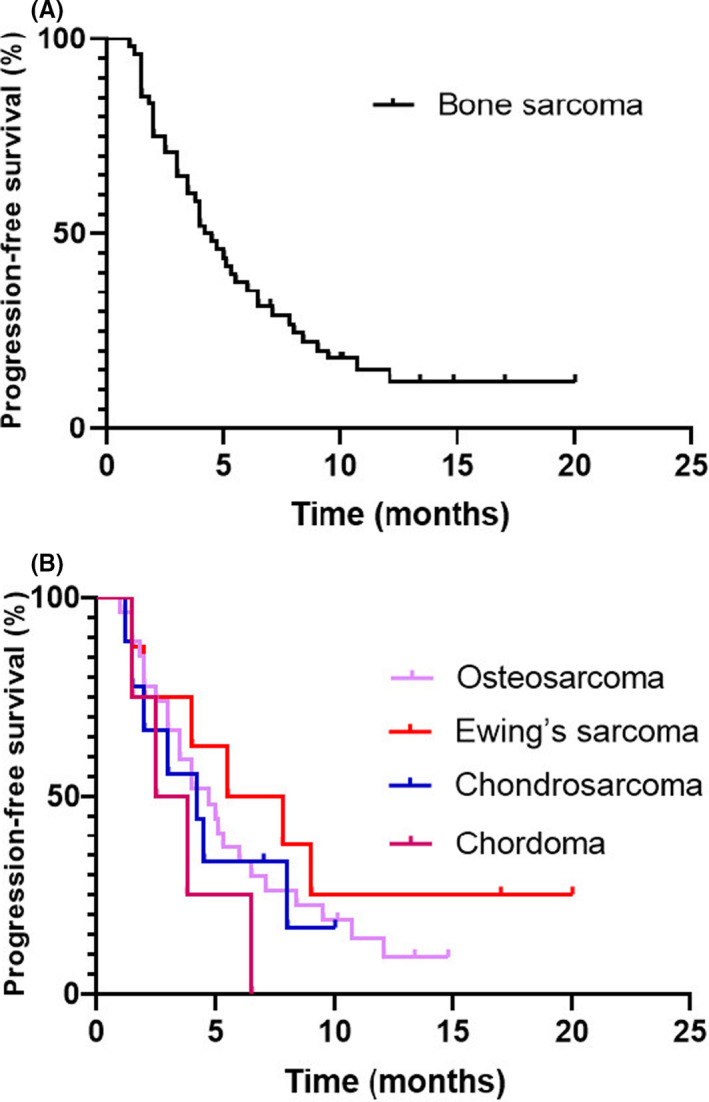
Kaplan–Meier curve of progression‐free survival (PFS). (A) PFS of 48 patients with unresectable or metastatic bone sarcoma. (B) PFS of patients with subtypes of bone sarcoma

For all patients with bone sarcoma, the median PFS was 4.6 months (95% CI: 1.3–16.6months), with a progression‐free rate (PFR) at 3 months of 70.8% and at 6 months of 35.4%. The median PFS varied among pathological subtypes: 4.7 months (95% CI: 1.3–13.9 months) for osteosarcoma, 6.7 months (95% CI: 1.6–21.1 months) for Ewing's sarcoma, 4.2 months (95% CI: 1.3–9.6 months) for chondrosarcoma, and 3.2 months (95% CI: 1.6–6.3 months) for chordoma (Table 2 and Figure 2).

### 3.4 Toxicity and safety

Anlotinib treatment was relatively well‐tolerated (Table 3), 44 (91.7%) of 48 patients experienced grade 1 or more AEs. The most frequent grade 1–2 treatment‐related AEs were hand‐foot syndrome (30, 62.6%), cholesterol elevation (21, 43.8%), hypertriglyceridemia (19, 39.6%), fatigue (17, 35.4%), proteinuria (17, 35.4%), hypertension (17, 35.4%). The grade 3/4 adverse events were not frequent following pneumothorax (2, 4.2%), hand‐foot syndrome (2, 4.2%), cholesterol elevation (1, 2.1%), hypertriglyceridemia (1, 2.1%), hypertension (1, 2.6%). Two patients developed pneumothorax that required anlotinib treatment discontinuation and pleural drainage with a chest tube. One of them permanently discontinued anlotinib because of recurrent pneumothorax. No deaths associated with AEs were reported.

**TABLE 3:** Adverse events (*N* = 48)

Adverse events	Total *n* (%)	Grade1/2 *n* (%)	Grade3/4 *n* (%)
Hand‐foot syndrome	30 (62.5)	28 (58.3)	2 (4.2)
Cholesterol elevation	21 (43.8)	20 (41.6)	1 (2.1)
Hypertriglyceridemia	19 (39.6)	18 (37.5)	1 (2.1)
Proteinuria	17 (35.4)	17 (35.4)	0 (0)
Fatigue	17 (35.4)	17 (35.4)	0 (0)
Hypertension	17 (35.4)	16 (33.3)	1 (2.1)
AST increased	16 (33.3)	16 (33.3)	0 (0)
ALT increased	16 (33.3)	16 (33.3)	0 (0)
Sore throat	12 (25)	12 (25)	0 (0)
TSH increased	12 (25)	12 (25)	0 (0)
Anorexia	12 (25)	12 (25)	0 (0)
Cough	11 (22.9)	11 (22.9)	0 (0)
Arthralgia	11 (22.9)	11 (22.9)	0 (0)
Diarrhea	10 (20.8)	10 (20.8)	0 (0)
Leukopenia	10 (20.8)	10 (20.8)	0 (0)
Voice alteration	9 (18.8)	9 (18.8)	0 (0)
Hypothyroidism	7 (14.6)	7 (14.6)	0 (0)
Pneumothorax	4 (8.3)	2 (4.2)	2 (4.2)

## 4 DISCUSSION

Bone sarcoma is a rare, heterogeneous family of malignancies with different histopathological and clinical features, constituting more than 10 histological subtypes. Despite significant advances in the development of multimodal therapies in recent years, the long‐term outcomes for patients with bone sarcoma unsuitable for surgery remain unfavorable.^2^, ^6^ To our knowledge, this study is the first to evaluate clinical outcomes of anlotinib in unresectable or metastatic bone sarcoma. Because bone sarcomas contain abundant bone matrix, even substantial anti‐tumor activity might not lead to significant tumor shrinkage.^10^ So, the ORR and median PFS were chosen as the dual primary endpoints. In this study, anlotinib showed promising antitumor activity in unresectable or metastatic bone sarcoma which was comparable to other TKIs, with the ORR of 10.4% (7.4% [2/27] for osteosarcoma, 37.5% [3/8] for Ewing's sarcoma, and 0% for chondrosarcoma and chordoma) and the median PFS of 4.6 months (4.7 months for osteosarcoma, 6.7 months for Ewing's sarcoma, 4.2 months for chondrosarcoma sarcoma, and 3.2 months for chordoma). AEs were mild and well‐tolerated. To determine the efficacy of anlotinib, histologically specific survival data should be further studied.

After more than 40 years, the treatment of osteosarcoma has presented a plateau. Outcomes of patients with recurrent or metastatic bone sarcoma are still dismal, with a 5‐year survival of less than 30%.^14^ Although the efficacy of several cytotoxic regimens, such as ifosfamide, etoposide, ifosfamide/etoposide, gemcitabine/docetaxel, is still lacking in prospective studies, their efficacy has been accepted and these regimens are widely used in clinical practice.^15^, ^16^, ^17^ The ORR of those therapies varies between 0% and 48% with a median PFS of about 3.5 months, Recent studies showed that several TKIs had clinical efficacy in advanced osteosarcoma in improved ORR of 10%–43.2% and prolonged median PFS of 4.5 months to 6.2 months. Xie and colleagues reported a series of 37 patients with advanced osteosarcoma treated with apatinib after failure of standard multi‐agent therapy in a Phase 2 trial. This was an interesting study in which the highest ORR of 43.2% was obtained to date, with a median PFS of 4.5 months.^18^ Sorafenib had an ORR of 8%, median PFS of 4 months, and 4‐month PFS of 46% in similar populations.^19^ Similar results of ORR of 7% and median PFS of 6 months were found by Longhi, who investigated pazopanib in 15 advanced osteosarcoma patients.^8^ A Phase 2 study of regorafenib in advanced osteosarcoma showed that the ORR was 13.6%, and the median PFS was 3.6 months.^20^ Most recently, in a Phase 2 study investigating cabozantinib, the ORR of 12% was reported in patients with advanced osteosarcoma, with a median PFS of 6.2 months.^10^ In this study, two patients achieved PR, 17 obtained SD. The ORR was 7.4% and the median PFS for advanced osteosarcoma patients was 4.7 months, the PFR at 3 months and 6 months were 72.9% and 35.4%, respectively. The results suggest that anlotinib achieved similar ORR to other TKIs except for apatinib. The median PFS seems to be comparable to that of other TKIs but slightly longer than regorafenib. Recently, Lagmay and colleagues recommended that if agent in advanced osteosarcoma could achieve a 16‐week PFS of more than 30%, it should be considered worthy of further investigation.^21^ According to this recommendation, the results of PFR at 6 months of 35.4% showed evidence of good antitumor activity of anlotinib in this setting.

Ewing's sarcoma is a family of tumors characterized by translocations generating the fusion between a gene encoding the EWSR1 and a gene encoding the ETS family of transcription factors.^22^ Standard therapy for localized disease is based on a combination of interval compression cytotoxic drugs, which is considered effective and well‐tolerated. However, the metastatic or relapsed disease is still a significant challenge, especially after the failure of standard chemotherapy with VDC or VDC‐IE. Cytotoxic drugs are widely used for advanced disease in clinical practice in this setting including cyclophosphamide and topotecan, temozolomide and irinotecan, docetaxel and gemcitabine, and high‐dose ifosfamide.^4^ Except for those drugs, insulin‐like growth factor I receptor (IGF‐1R) inhibitors and small molecular TKIs are the most well‐studied targeted drugs. The efficacy of the targeted drugs was encouraging, with an ORR of 0–26% and a median PFS of 1.9 months to 7.9 months. A Phase 2 study of ganitumab showed that an ORR of 5% and a median of 7.9 months were obtained in 22 patients with Ewing's family tumor.^23^ A recent study showed that cabozantinib obtained a high ORR of 26% (10 of 39 patients) and a median PFS of 4.4 months.^10^ All patients in this study had distant metastases and tumor progression after standard chemotherapy treatment, three patients had PR, the ORR was 37.5%, and the median PFS was 6.7 months. A patient had tumor shrinkage of 50% with PFS of 12 months, and another had tumor shrinkage of 70% with PFS of 20 months. Four patients got SD for more than 4 months, which indicated that anlotinib might be a potentially effective drug in advanced Ewing's sarcoma.

Due to the diverse biological behaviors and treatment of bone sarcoma, the clinical outcomes of advanced diseases are different. Conventional chondrosarcomas and chordomas are marked by low sensitivity to conventional chemotherapy and radiotherapy. However, a retrospective study of 171 patients with locally advanced chondrosarcomas showed that adriamycin‐based chemotherapy or noncytotoxic chemotherapy may prolong median OS compared with untreated patients (20 months vs. 15 months).^24^ Recent studies showed that TKIs achieved diverse efficacy in cases or small sample studies. A Phase 2 trial showed that pazopanib achieved a disease control rate (DCR) at 16 weeks of 43% and a median PFS of 7.9 months in 43 patients with advanced chondrosarcoma.^26^ Nine patients were included in this study, of whom five had lung metastasis. Although no CP or PR was observed, four patients had SD. The median PFS was 4.2 months, lower than that of pazopanib, dasatinib, and sirolimus combined with other drugs,^25^ suggesting that anlotinib monotherapy achieved limited anti‐tumor effect on patients with advanced chondrosarcoma. There were only four patients with chordoma in this study, with a median PFS of 3.2 months.

Anlotinib for bone sarcoma obtained encouraging clinical results in this study, similar to other TKIs presented in previous studies, especially for osteosarcoma and Ewing's sarcoma. Preclinical studies have shown that TKIs could improve vascular normalization, overcome therapeutic resistance and increase the anti‐tumor effect. So, it may be reasonable to combine anlotinib with chemotherapy to improve the clinical outcomes further. To date, there are two clinical trials with anlotinib in bone sarcoma patients. The first is an ongoing Phase 2 trial which aims to assess the concurrent use of chemotherapy with anlotinib for the treatment of stage IIB classical osteosarcoma of the extremities. The primary endpoint is a 24‐month event‐free survival rate, and the results would be released in early 2025(ChiCTR 2000033298).^26^ The second is a Phase 1/2 trial which enrolled 41 patients to assess the efficacy and safety of vincristine, irinotecan, and anlotinib in patients with unresectable Ewing's sarcoma. The primary endpoint for Phase 1b is to determine irinotecan dose for Phase 2, and the Phase 2 primary endpoint is ORR at 12 weeks (ORR_12w_). The results concluded that an irrnotecan‐based regimen combined with anlotinib showed a promising clinical efficacy, with ORR_12w_ of 62.5% for adults and 83.3% for children with advanced Ewing's sarcoma.(NCT 03416517).^27^

Treatment‐related AEs were observed in this study, comparable to those in previous studies of other cancers. No new AEs related to anlotinib were found. AEs were typically graded 2 or lower, and AEs of grade 3 could be generally managed by reducing the dose or suspending treatment temporarily. Hypertension was not as severe and slightly less than what was reported in other literature, which may be because of the young population. The most frequent grade 1–2 AEs were consistent with other reports, followed by hand‐foot syndrome, cholesterol elevation, hypertriglyceridemia, fatigue, proteinuria, hypertension. The most frequent grade 3–4 AEs were pneumothorax and hand‐foot syndrome. One patient with pneumothorax stopped treatment permanently because of two relapses, which may be related to subpleural and pleural metastasis and cavitary lung lesions after treatment. Five patients needed to reduce the dose due to AEs, three of whom were under 18 years old, so the appropriate dose for adolescents need to be further explored in adolescent patients.

This study had some limitations. First, this study had the characteristics of a retrospective design and a small sample size. It would be necessary to investigate the clinical benefits and safety of anlotinib for this disease in prospective randomized controlled studies. Second, we did not further classify the subtypes of osteosarcoma and chordoma because of the rarity and various types of bone sarcoma. Further analysis with a large number of patients with histological‐specific subtype of bone sarcoma would be needed. Third, we did not analyze the prognostic factors of the efficacy of anltonib in this study. Thus, it would be necessary to analyze tumor subtypes or some biomarkers to stratify patients to determine anlotinib treatment in the future.

In conclusion, anlotinib resulted in favorable anti‐tumor activity in terms of ORR and median PFS in patients with unresectable or metastatic bone sarcoma, suggesting that anlotinib may be a potential option for patients with advanced bone sarcoma. Most AEs are mild and manageable. The study of anlotinib in some particular subtypes of bone sarcoma, such as advanced osteosarcoma and Ewing's sarcoma, warrants further investigation.

## ETHICS STATEMENT

The study was approved by the Institutional Review Board of The Affiliated Cancer Hospital of Zhengzhou University, Zhengzhou Orthopedics Hospital and Henan Provincial People's Hospital, and conducted in line with the established guidelines, consistent with the Declaration of Helsinki. After the purpose and aim of this study were fully explained, written informed consents were gained from each patient or legal guardian of a given patient before treatment.

## CONFLICT OF INTEREST

All authors declare no competing interests.

## Data Availability

All data generated or analyzed during this study are included in this published article.
